# Sharing quality and safety improvement work in the field of mental health

**DOI:** 10.1192/bjb.2018.3

**Published:** 2018-02

**Authors:** Amar Shah

**Affiliations:** Consultant Forensic Psychiatrist, East London NHS Foundation Trust, and Quality Improvement Lead, Royal College of Psychiatrists; email: amarshah@nhs.net

I welcome the findings of D'Lima, Crawford, Darzi and Archer^1^ on the relative scarcity of publications related to mental health quality and safety improvement within reputable quality and safety journals.

I agree with the authors’ proposition that there is increasing interest in the application of improvement science within the mental health field. A large number of providers of mental health services in the UK and beyond are now starting to apply quality improvement methods at scale. The Royal College of Psychiatrists has established a quality improvement committee over the past year, and has recently appointed its first quality improvement lead. There is also an organically growing global mental health improvement network (#MHimprove), which meets twice a year and has begun to present and share knowledge at large international conferences.

As both the College quality improvement lead and the lead for quality at East London NHS Foundation Trust (ELFT), with perhaps one of the largest improvement programmes in the world within mental health services, my experience agrees with the conclusion of the authors that publishing mental health improvement work within reputable quality and safety journals is a struggle. Our efforts to share real-world improvement work have largely been unsuccessful in the high-quality journals within this field. My theory, both as a submitting author and a reader of these journals, is that the journals are still focusing more on the research and evaluation end of the spectrum, as opposed to real-world, messy improvement work in mental health services.

As an example, the use of Shewhart (control) charts to demonstrate improvement over time, which is seen as best practice by improvers across the globe, is still frowned upon by journals (both subject-matter specialist journals and quality and safety journals), who prefer enumerative statistics in the form of pre- and post-comparison of averages. This jars with the real world of applied science, where there is no pre- and post-state, but a gradual and iterative transition, fuelled by multiple tests of change with increasing degree of belief and reliability in the change package.

Despite these challenges, ELFT has published approximately 15 peer-reviewed articles over the past 4 years and has 3–4 articles continuously in the process of submission. All published papers are made available transparently to everyone through the ELFT quality improvement website (https://qi.elft.nhs.uk), which has now had over half a million hits in the past 3.5 years. Going even further, ELFT aims to share learning from all completed projects on its website, in acknowledgement of the fact that practising improvers and clinicians will always struggle to find time to publish all completed improvement work in peer-reviewed journals.

This brings me to the question: are journals still relevant in a world of fast-paced knowledge-sharing and acquisition, and with increasingly digital and connected networks? More than 500 leaders and clinicians from more than 50 different provider organisations have now been to visit ELFT to find out more first-hand about the quality improvement work taking place. At ELFT, we are also using the web and social media to share knowledge in real time and transparently with everyone, as we recognise our responsibility in helping to grow the field of knowledge within mental health improvement, and also to foster confidence in the use of quality improvement.

So, while my personal experience leads me to agree with the authors that publishing mental health improvement in journals remains challenging and often puts people off from even submitting, I would also suggest that there is much more active improvement work taking place and being shared by mental health services globally than might be apparent from looking at high-quality peer-reviewed journals alone.
